# Management of Hepatic Visceral Crisis Using Chemoimmunotherapy in PD-L1-High Metastatic Triple-Negative Breast Cancer: A Case Report

**DOI:** 10.3390/diagnostics16060924

**Published:** 2026-03-20

**Authors:** Larisa Maria Badau, Paul Epure, Madalin-Marius Margan, Andrei Dorin Ciocoiu, Gabriel-Mugurel Dragomir, Brigitha Vlaicu

**Affiliations:** 1 Doctoral School in Medicine, “Victor Babes” University of Medicine and Pharmacy, Eftimie Murgu Square No. 2, 300041 Timisoara, Romania; larisa.badau@umft.ro; 2 Department of Oncology, ONCOHELP Hospital Timisoara, Ciprian Porumbescu Street, No. 59, 300239 Timisoara, Romania; 3 Discipline of Hygiene, “Victor Babes” University of Medicine and Pharmacy, Eftimie Murgu Square No. 2, 300041 Timisoara, Romania; vlaicu@umft.ro; 4 “Pius Brinzeu” County Emergency Hospital, 300723 Timisoara, Romania; epurepaul1297@gmail.com; 5 Department of Public Health and Sanitary Management, “Victor Babes” University of Medicine and Pharmacy, Eftimie Murgu Square No. 2, 300041 Timisoara, Romania; 6 Department of Microscopic Morphology, “Victor Babes” University of Medicine and Pharmacy, 300041 Timisoara, Romania; andrei.ciocoiu@umft.ro; 7 Department of Oncology, City Clinical Emergency Hospital of Timisoara, “Victor Babes”, Blvd. No. 22, 300595 Timisoara, Romania; 8 Department of Teaching Training, Politehnica University of Timisoara, 300006 Timisoara, Romania; mugur.dragomir@upt.ro

**Keywords:** visceral crisis, immunotherapy, metastatic triple-negative breast cancer

## Abstract

**Background/Objectives:** Patients with metastatic breast cancer and visceral crisis are systematically excluded from clinical trials, leaving clinicians without evidence-based therapeutic guidance. To the best of our knowledge, no published reports have described the use of combined chemo-immunotherapy in mTNBC complicated by hepatic visceral crisis. **Case presentation:** We report the case of a 45-year-old woman with PD-L1-high recurrent TNBC who presented with acute, life-threatening hepatic failure. Laboratory evaluation revealed marked transaminase elevation, cholestasis, rising bilirubin levels, and clinical deterioration consistent with hepatic visceral crisis. Due to severe hepatic impairment, a sequential therapeutic strategy was adopted: treatment was initiated with dose-reduced weekly paclitaxel (80% of the standard dose), and pembrolizumab (200 mg every three weeks) was introduced at the fourth cycle. Shortly after immunotherapy initiation, the patient experienced rapid clinical improvement accompanied by significant biochemical recovery and radiologic tumor regression. Although disease progression occurred after four months, hepatic visceral crisis did not recur. **Discussion:** This case questions the conventional restriction of immunotherapy in the setting of severe hepatic dysfunction. The rapid biochemical recovery observed after pembrolizumab initiation suggests that immunologic antitumor activity may be preserved despite significant hepatic impairment. Furthermore, the high PD-L1 expression in this patient indicates that its predictive value may extend even to the context of visceral crisis. **Conclusions:** Our findings suggest that immunotherapy in combination with chemotherapy may represent a feasible therapeutic strategy in selected patients with PD-L1-high mTNBC presenting with hepatic visceral crisis.

## 1. Introduction

Breast cancer is the most common malignancy among women worldwide [1]. However, significant discrepancies persist in cancer epidemiology across European countries, with substantial variations observed in cancer incidence and mortality [2]. Breast cancer incidence in Romania is rising, making it the second leading cause of cancer-related mortality after lung cancer, partly due to the lack of a national mammography screening program [3].

Among the distinct molecular subtypes, triple-negative breast cancer (TNBC), defined by the lack of estrogen (ER), progesterone (PR) and human epidermal growth factor receptor 2 (HER2) expression, accounts for 15–20% of all cases, and is more frequently diagnosed in younger women [4]. TNBC is characterized by an aggressive biological phenotype, a tendency toward early visceral dissemination and an overall poorer prognosis compared with hormone receptor-positive (HR+) or HER2-positive breast cancer subtypes [5]. In early-stage TNBC, achieving a pathological complete response (pCR) remains a key prognostic indicator. TNBC tumors are approximately three times more likely to develop distant metastases within the first five years [6]. When relapse occurs, it frequently involves visceral organs and is often associated with a rapid and clinically significant deterioration [7].

Until recently, therapeutic advances in TNBC lagged behind those of other breast cancer subtypes, due to the lack of actionable targets, leaving patients with limited therapeutic options. In metastatic triple-negative breast cancer (mTNBC), the therapeutic landscape has undergone substantial transformation with the introduction of immune checkpoint inhibitors (ICIs), particularly pembrolizumab, which has broadened treatment options and improved clinical outcomes of TNBC patients. The phase III KEYNOTE-355 trial demonstrated that the addition of pembrolizumab to standard chemotherapy (Paclitaxel, Nab-Paclitaxel, or Gemcitabine/Carboplatin) significantly improved both progression-free survival (PFS) and overall survival (OS) in patients with PD-L1 (Programmed death-ligand 1) CPS (Combined Positive Score) ≥ 10, thereby establishing this combination as a first-line therapeutic standard in this subgroup [8].

However, it is important to acknowledge that large randomized phase III trials, such as KEYNOTE-355, generally require adequate organ function for study enrollment. Consequently, patients presenting in visceral crisis (VC), defined clinically as severe organ dysfunction, which is assessed based on clinical signs, symptoms, laboratory abnormalities, and rapid disease progression, are often excluded from these studies [9]. VC may occur in up to 60% of patients with visceral metastases, although TNBC accounts for only a minority of cases in some reported cohorts [9,10]. Hepatic involvement is the most frequent presentation, accounting for approximately 30–50% of cases, followed by pulmonary involvement [9]. The type of VC has also been identified as an independent prognostic factor, with bone marrow involvement associated with better outcomes and hepatic involvement with the poorest prognosis [11]. Management in this setting remains challenging and largely empirical, often necessitating the rapid initiation of cytotoxic therapy to achieve prompt disease control and reverse organ dysfunction before immunotherapy can exert its clinical benefit.

This case report describes a 45-year-old woman with recurrent TNBC who presented with acute, life-threatening hepatic failure. Treatment was initiated with paclitaxel, followed by the subsequent addition of pembrolizumab, leading to remarkable clinical and biochemical improvement despite the frank hepatic VC at presentation.

## 2. Case Presentation

A healthy 42-year-old premenopausal woman with normal body mass index presented to our Oncology Department in July 2021 with an established histopathological diagnosis of breast carcinoma. Her family history was significant for ovarian cancer in her mother, who died at the age of 59.

A core needle biopsy of a right breast mass was initially performed but yielded inconclusive results for malignancy. Consequently, excision of level I right axillary lymph nodes was undertaken for definitive pathological assessment. Pathological examination revealed a high-grade (G3), poorly differentiated invasive carcinoma of no special type (NST), with 18 metastatic lymph nodes identified. Immunohistochemical analysis demonstrated a triple-negative phenotype, with absence of ER, PR, and HER2 expression. The Ki-67 proliferation index was 50%, indicating a highly proliferative tumor. An ulterior whole-body computed tomography (CT) did not show evidence of distant metastasis. The stage was determined to be cT1c pN3 cM0, stage IIIC based on the eighth edition of the Cancer Staging Manual for Breast Cancer by the American Joint Committee on Cancer. Germline *BRCA1/2* testing was performed on peripheral blood using next-generation sequencing (NGS) and multiplex ligation-dependent probe amplification (MLPA), and no pathogenic variants or genomic rearrangements were identified.

The patient received eight cycles of neoadjuvant dose-dense chemotherapy, consisting of epirubicin and cyclophosphamide followed by docetaxel, administered between August and November 2021. In December 2021, the patient underwent a right radical mastectomy with immediate breast reconstruction using a tissue expander. Postoperative pathological examination revealed residual intermediate-grade ductal carcinoma in situ (DCIS), consistent with pCR. Notably, the residual lesion demonstrated receptor discordance compared with the initial biopsy, exhibiting strong hormone receptor expression (ER 100%, PR 70%), while remaining HER2-negative. The Ki-67 proliferation index was markedly reduced to 1%. The patient received adjuvant radiotherapy (two grays daily, 25 fractions) to the right axilla. Given the newly identified hormone receptor positivity, adjuvant hormone therapy with Anastrozole at 1 mg daily was initiated alongside ovarian suppression with goserelin (3.6 mg every 28 days).

**Figure 1:**
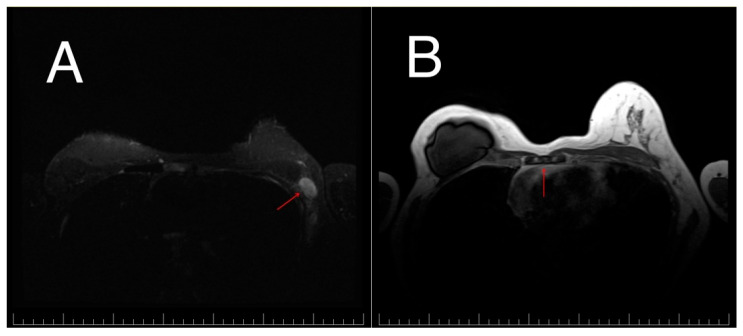
Axial contrast-enhanced breast MRI scan at the time of metastatic recurrence. (**A**) Left axillary lymphadenopathy. (**B**) Bone metastases involving the sternum.

**Figure 2:**
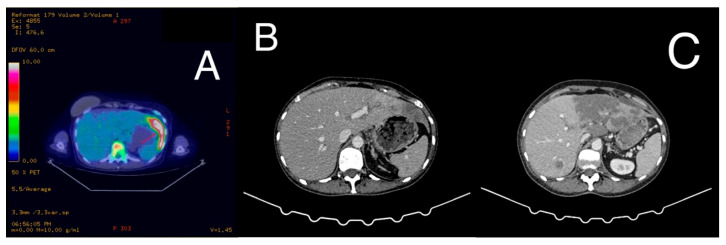
Radiologic assessment of hepatic metastasis before and during treatment. (**A**) Baseline 18F-FDG PET-CT showing intense metabolic activity within the left hepatic lobe metastasis. (**B**) Contrast-enhanced CT performed three months after initiation of chemoimmunotherapy demonstrating a reduction in hepatic metastasis size. (**C**) Subsequent CT revealing enlargement and new confluent hepatic lesions.

The patient remained disease-free for approximately 28 months. In April 2024, routine surveillance mammography identified a suspicious left axillary lymph node, accompanied by an elevated Cancer Antigen 15-3 (CA 15-3) level, 42.16 U/mL. Subsequent breast magnetic resonance imaging (MRI) confirmed left axillary lymphadenopathy and revealed multiple sternal lesions suspicious for bone metastases (Figure 1A,B). A positron emission tomography–computed tomography (PET-CT) scan was promptly performed, revealing extensive hypermetabolic activity in multiple lymph node stations, including laterocervical, axillary, mediastinal, and retroperitoneal regions. Additionally, an 84 × 30 mm hypermetabolic lesion was identified in hepatic segment II, along with widespread skeletal metastases (Figure 2A). An excisional biopsy of the left axillary adenopathy confirmed metastatic carcinoma of breast origin. Immunohistochemical analysis demonstrated reversion to a triple-negative phenotype (ER 0%, PR 0%, HER2 0), with a Ki-67 proliferation index of 40%. Given the recurrence of the disease, PD-L1 expression was assessed, revealing a markedly elevated CPS of 80 using the 22C3 clone.

During the diagnostic workup, the patient’s clinical status worsened by developing symptoms consistent with hepatic VC, such as progressive upper abdominal pain, abdominal distension, mild dyspnea, anorexia, and vomiting. Physical examination revealed marked hepatomegaly. Laboratory evaluation demonstrated severe hepatocellular injury accompanied by cholestasis, with aspartate aminotransferase (AST) 1129.72 U/L, alanine aminotransferase (ALT) 325 U/L, gamma-glutamyl transferase (GGT) 1119.34 U/L, and alkaline phosphatase (ALP) 444.55 U/L. Notably, total bilirubin (TBIL) remained within normal limits at that time, but CA 15-3 had increased to 146 U/mL. Viral and autoimmune etiologies of liver injury were excluded. Imaging revealed no intrahepatic or extrahepatic biliary dilation. Supportive treatment, including hepatoprotective therapy, was administered during this period. At this stage, the patient fulfilled clinical criteria consistent with hepatic VC based on rapidly worsening liver function tests, symptomatic hepatomegaly, and clinical deterioration.

Given the diagnosis of PD-L1-positive recurrent TNBC presenting with VC, the standard first-line chemo-immunotherapy regimen was carefully considered as the initial therapeutic approach. However, due to the patient’s severely impaired hepatic function, a sequential strategy was adopted, with chemotherapy initiated first to allow close clinical monitoring before the introduction of immunotherapy. This approach was chosen despite the limited evidence in the literature regarding the safety and efficacy of ICIs in the case of significant hepatic dysfunction. Thus, in June 2024, treatment was initiated with weekly paclitaxel at a dose reduced to 80% of the standard 90 mg/m^2^. Concurrently, bisphosphonate therapy with ibandronic acid was started for the management of bone metastases.

**Figure 3:**
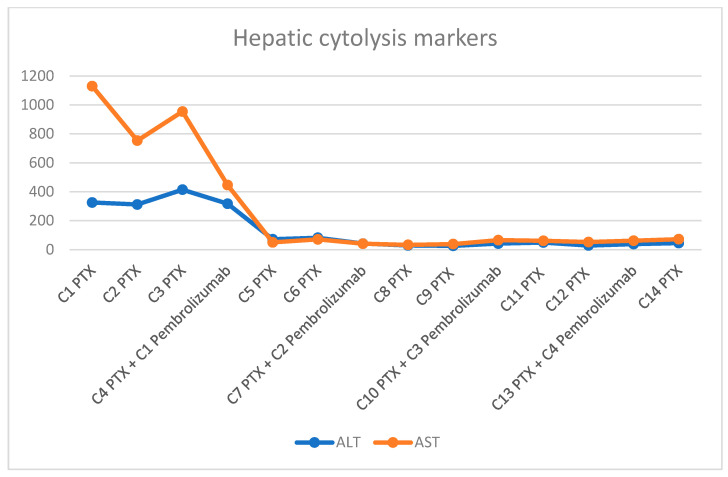
Changes in transaminase levels following the introduction of pembrolizumab.

**Figure 4:**
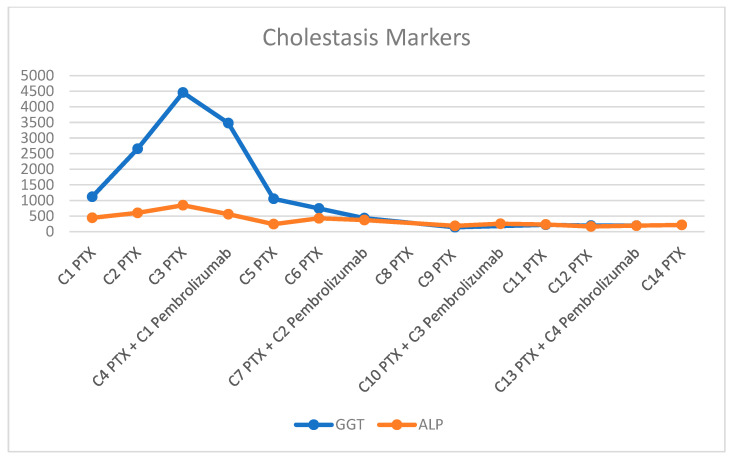
Changes in GGT and ALP levels following the introduction of pembrolizumab.

**Figure 5:**
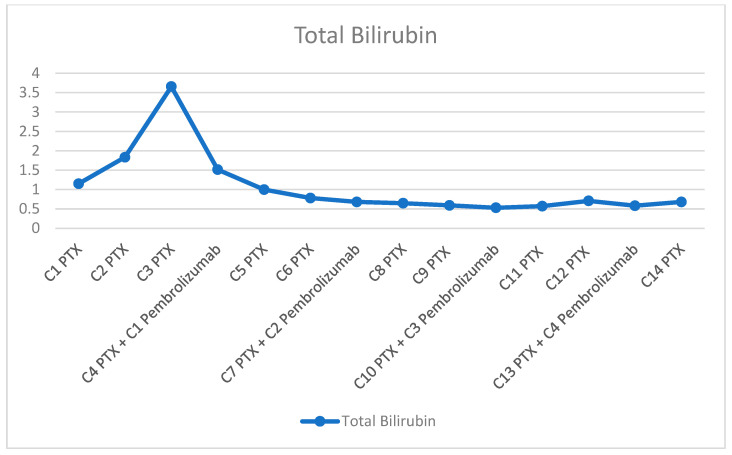
Changes in TBIL levels following the introduction of pembrolizumab.

Despite initial therapy, hepatic dysfunction progressively worsened, with peak laboratory values by the third administration of paclitaxel (AST 954 U/L, ALT 414.19 U/L, GGT 4458.03 U/L, ALP 847.16 U/L, TBIL 3.655 mg/dL). This clinical decline was accompanied by progressive abdominal distension due to tense ascites requiring therapeutic paracentesis, as well as massive bilateral lower extremity edema. However, in this life-threatening clinical context, pembrolizumab (200 mg every three weeks) was added at the time of the fourth paclitaxel administration. Following its initiation, prior to her 5th cycle, the patient experienced progressive clinical improvement accompanied by significant biochemical recovery (AST 82.06 U/L, ALT 70.91 U/L). CA 15-3 levels declined to 61.53 U/mL. Consequently, paclitaxel was escalated to the full standard dose. The number of treatment cycles and the trends in liver function tests are illustrated in Figure 3, Figure 4 and Figure 5. After eight cycles of paclitaxel and two cycles of pembrolizumab, imaging reassessment by CT showed a decrease in the size of the hepatic metastasis (Figure 2B). Similarly, a marked regression of nodal disease was observed, with the left axillary conglomerate decreasing in size from 34 mm to 21 mm. Also, the skeletal metastases demonstrated increasing osteosclerosis, consistent with a treatment-related response. Treatment with pembrolizumab was complicated by immune-related hypothyroidism, managed with levothyroxine replacement therapy at a dose of 25 µg daily.

After 14 cycles of paclitaxel and 4 cycles of pembrolizumab, follow-up imaging demonstrated a mixed response, characterized by continued regression of lymphadenopathy, but progression of visceral and bone metastases. New confluent hepatic lesions were observed, replacing much of the left hepatic lobe (Figure 2C), and an increased number of bone metastases. Importantly, at the time of radiologic progression, the patient did not exhibit hepatic VC or severe hepatic dysfunction. Second-line therapy with sacituzumab govitecan (10 mg/kg) was initiated. However, the second cycle was complicated by Clostridium Difficile-associated enterocolitis and subsequent sepsis, which ultimately resulted in the patient’s death in December 2024.

## 3. Discussion

VC represents one of the most challenging clinical scenarios in the management of metastatic breast cancer (MBC), characterized by severe organ dysfunction, rapidly progressive disease, and an imminent risk of life-threatening deterioration. The concept of VC has remained unchanged since the 5th International Consensus Conference for Advanced Breast Cancer (ABC5) [12,13]. Its real-world prevalence is uncertain; however, it is estimated to occur in approximately 10–20% of patients with MBC [9,12,14]. Although VC would be expected to disproportionately affect aggressive breast cancer subtypes, TNBC represented only 11% of cases in a reported study, despite its well-established association with early visceral dissemination [10].

Several cohort studies have demonstrated heterogeneity in the clinical presentation of VC, with the liver representing one of the most frequently affected organs. In the cohort reported by Yang et al., approximately 50% of patients experienced VC secondary to hepatic involvement, 25% due to bone marrow infiltration, 15% from meningeal disease, and 7% as a result of pulmonary involvement [11]. Sbitti et al. reported a cohort of 35 patients presenting with VC, of whom 55% had hepatic dysfunction and 35% developed respiratory failure [15]. The prevalence of hepatic and lung involvement was further confirmed in a larger study including 261 patients with VC, in which 51% presented with liver failure and 17% with pulmonary lymphangitic carcinomatosis [16]. According to the 2020 European School of Oncology (ESO)—European Society of Medical Oncology (ESMO) ABC5 guidelines, hepatic VC is defined based on clinical context and laboratory findings as a rapid increase in bilirubin > 1.5 times the upper limit of normality, in the absence of Gilbert’s syndrome or biliary tract obstruction [12].

In routine clinical practice and according to current guideline recommendations, chemotherapy remains the mainstay of treatment in VC, primarily because it is expected to induce a more rapid tumor response in the setting of extensive and aggressive metastatic disease. However, these recommendations are largely based on expert consensus rather than prospective clinical trial data, as patients with VC are generally excluded from most trials, due to concerns regarding treatment tolerance and the risk of severe toxicity in the context of organ dysfunction. This systematic exclusion is exemplified by the KEYNOTE-355 trial, whose eligibility criteria required adequate organ function at study entry. As a potential therapeutic strategy, combining ICIs with chemotherapy may elicit a faster clinical response compared to the typical delayed onset of ICIs monotherapy.

When comparing our patient’s clinical course with the outcomes reported in the KEYNOTE-355 trial, several important distinctions become apparent. In KEYNOTE-355, patients with PD-L1 CPS ≥ 10 treated with pembrolizumab plus chemotherapy achieved a median PFS of 9.7 months (HR: 0.66; 95% CI: 0.50 to 0.88) and a median OS of 23.0 months (HR: 0.73, 95% CI: 0.55–0.95, *p* = 0.0185) [8]. In contrast, our patient, who would have been ineligible for trial enrollment due to severe hepatic dysfunction, experienced reversal of hepatic VC and meaningful clinical stabilization, with a PFS of 4 months before disease progression required second-line therapy. Her OS of 8 months from the initiation of first-line treatment, although shorter than the outcomes reported in the trial population, remains noteworthy given the dismal prognosis historically associated with hepatic VC, where median survival has been reported at approximately 4.7 weeks [15]. These findings suggest that PD-L1 expression could retain its predictive value even in the context of VC, supporting the consideration of ICIs in carefully selected patients.

The guidelines do not specify an optimal chemotherapy regimen for patients presenting with VC, and treatment selection is frequently complicated by clinical frailty and underlying organ impairment. In a real-world cohort reported by Andrade et al., paclitaxel, gemcitabine, cisplatin, carboplatin, and doxorubicin were among the most commonly used agents, administered either as monotherapy or as part of combination chemotherapy regimens. The selection of a chemotherapy regimen in the context of severe hepatic impairment requires careful consideration of drug pharmacokinetics and hepatotoxicity profiles. We initiated treatment with dose-reduced weekly paclitaxel, with subsequent escalation to the full standard dose for several reasons. First, paclitaxel is considered to have a relatively more manageable hepatic safety profile compared with anthracyclines and certain platinum-based regimens, which may be contraindicated in the setting of significant hepatic dysfunction [17]. Second, the weekly administration of paclitaxel permits more frequent clinical and laboratory monitoring, providing flexibility to withhold or further adjust doses according to tolerability and evolving hepatic function. Moreover, in the prior dose-dense neoadjuvant setting, the patient had tolerated docetaxel without significant hepatotoxicity, supporting the feasibility of a taxane-based approach despite the presence of hepatic VC. The addition of immunotherapy with pembrolizumab in this context was both challenging and unconventional. ICIs are primarily cleared through proteolytic catabolism rather than hepatic cytochrome P450 metabolism, theoretically rendering their pharmacokinetics less susceptible to impairment in the setting of hepatic dysfunction [18]. However, severe hepatic dysfunction may significantly alter immune homeostasis and potentially increase the risk of immune-related adverse events, particularly immune-mediated hepatitis. Evidence regarding the use of ICIs patients with baseline grade 3–4 hepatic impairment remains scarce. Moreover, the available literature predominantly describes cases involving underlying chronic liver disease, rather than acute hepatic failure secondary to extensive metastatic infiltration [19,20]. Our decision to delay the initiation of pembrolizumab until the fourth cycle of chemotherapy was intended to allow for initial disease control and careful clinical reassessment, with the aim of limiting potential additive hepatotoxicity before introducing immunotherapy. The observed clinical and biochemical improvement may have also been influenced by the cytoreductive effect of weekly paclitaxel and supportive care measures.

The occurrence of immune-related adverse events, specifically hypothyroidism, was consistent with the established safety profile of ICIs and did not appear to be exacerbated by the underlying hepatic dysfunction. Hypothyroidism has been reported in approximately 11.5% of patients treated with ICIs and is generally manageable with thyroid hormone replacement therapy [21]. Importantly, the patient did not develop immune-related hepatitis, which had represented a major concern given the severity of her baseline hepatic impairment.

An additional noteworthy aspect of this case is the discordance in receptor status throughout the disease course. While the initial core biopsy demonstrated a triple-negative phenotype, the post-neoadjuvant surgical specimen revealed residual ductal carcinoma in situ with positive hormone receptor expression. However, biopsy of the subsequent metastatic recurrence once again confirmed a triple-negative profile. This dynamic shift underscores the well-recognized intratumoral and intertumoral heterogeneity of breast cancer and may also reflect treatment-related clonal selection. Receptor discordance between primary and residual or metastatic lesions has been increasingly recognized, with reported rates of approximately 40% in breast cancer [22,23]. These findings emphasize the critical importance of repeat rebiopsy at the time of metastatic recurrence to accurately guide subsequent treatment decisions.

To our knowledge and based on a focused literature search, there are no published cases reporting the use of combined chemo-immunotherapy in mTNBC complicated by hepatic VC. Although VC traditionally favors the initiation of chemotherapy to achieve rapid disease control, this case suggests that the addition of ICIs may be feasible in carefully selected patients. In our patient, liver function tests and clinical status did not improve during the initial cycles of paclitaxel and began to normalize only after the introduction of pembrolizumab. However, given the sequential administration of therapies and the uncontrolled nature of a single case, the relative contribution of chemotherapy, immunotherapy, and supportive care cannot be definitively determined.

In our review of the literature, we found that clinical data on MBC presenting with VC are largely derived from cohorts of patients with HR+/HER2-negative disease. In this context, CDK4/6 inhibitors represent one of the newer therapeutic agents demonstrating efficacy in this subgroup [24,25,26]. Despite therapeutic advances in HER2-positive/low TNBC with antibody–drug conjugates (ADC) and ICIs, data on their efficacy and safety in the setting of VC remain limited, as these patients are underrepresented in clinical trials [27,28,29].

## 4. Conclusions

Our findings suggest that a sequential strategy consisting of initial chemotherapy followed by the introduction of immunotherapy may represent a feasible treatment approach for mTNBC presenting with VC and severe hepatic dysfunction. The marked hepatic improvement observed after immunotherapy initiation supports its potential role in disease control in this setting. Notably, the markedly elevated PD-L1 expression in this case supports its potential predictive value even in the setting of VC, reinforcing the rationale for incorporating ICIs in carefully selected patients. There are no published data describing the successful treatment of hepatic VC in TNBC using ICIs combined with chemotherapy. The experience described in this report may contribute to evolving clinical practice and inform future therapeutic strategies in this life-threatening condition, although the findings should be considered hypothesis-generating rather than evidence supporting the efficacy or safety of chemoimmunotherapy in this setting. Until such data become available, treatment decisions should be individualized, taking into account tumor biology, degree of organ dysfunction, performance status, and realistic goals of care.

## Data Availability

The data presented in this study are available upon request from the corresponding author.

## Abbreviations

The following abbreviations are used in this manuscript:

TNBC	Triple-Negative Breast Cancer
ER	Estrogen receptor
PR	Progesteron receptor
HER2	Human epidermal growth factor receptor 2
HR+	Hormone receptor-positive
pCR	Pathological complete response
mTNBC	Metastatic triple-negative breast cancer
ICIs	Immune checkpoint inhibitors
PD-L1	Programmed death-ligand 1
CPS	Combined Positive Score
PFS	Progression-free survival
OS	Overall survival
VC	Visceral crisis
NST	No special type
CT	Computed tomography
NGS	Next-generation sequencing
MLPA	Multiplex ligation-dependent probe amplification
DCIS	Ductal carcinoma in situ
CA 15-3	Cancer Antigen 15-3
MRI	Magnetic resonance imaging
PET-CT	Positron emission tomography–computed tomography
AST	Aspartate aminotransferase
ALT	Alanine aminotransferase
GGT	Gamma-glutamyl transferase
ALP	Alkaline phosphatase
TBIL	Total bilirubin
MBC	Metastatic breast cancer
ABC5	5th International Consensus Conference for Advanced Breast Cancer
ESO	European School of Oncology
ESMO	European Society of Medical Oncology
